# Platelet degranulation and bleeding phenotype in a large cohort of Von Willebrand disease patients

**DOI:** 10.1111/bjh.18145

**Published:** 2022-03-22

**Authors:** Maurice Swinkels, Ferdows Atiq, Petra E. Bürgisser, Iris van Moort, Karina Meijer, Jeroen Eikenboom, Karin Fijnvandraat, Karin P. M. van Galen, Joke de Meris, Saskia E. M. Schols, Johanna G. van der Bom, Marjon H. Cnossen, Jan Voorberg, Frank W. G. Leebeek, Ruben Bierings, A. J. Gerard Jansen, WiN study group, K. Fijnvandraat, M. Coppens, J. de Meris, L. Nieuwenhuizen, K. Meijer, R. Y. J. Tamminga, P. F. Ypma, H. C. J. Eikenboom, J. G. van der Bom, F. J. W. Smiers, B. Granzen, F. Moenen, P. Brons, S. E. M. Schols, F. W. G. Leebeek, M. H. Cnossen, F. Atiq, C. B. van Kwawegen, K. P. M. van Galen

**Affiliations:** ^1^ Department of Hematology Erasmus University Medical Center Rotterdam The Netherlands; ^2^ Department of Hematology, University Medical Center Groningen University of Groningen Groningen The Netherlands; ^3^ Department of Internal Medicine, Division of Thrombosis and Hemostasis Leiden University Medical Center Leiden The Netherlands; ^4^ Einthoven Laboratory for Vascular and Regenerative Medicine Leiden University Medical Center Leiden The Netherlands; ^5^ Department of Pediatric Hematology Emma Children's Hospital‐Academic Medical Centre Amsterdam The Netherlands; ^6^ Van Creveldkliniek, University Medical Center Utrecht Utrecht University Utrecht The Netherlands; ^7^ Netherlands Hemophilia Society Leiden The Netherlands; ^8^ Department of Hematology Radboud University Medical Center and Hemophilia Treatment Center Nijmegen‐Eindhoven‐Maastricht Nijmegen The Netherlands; ^9^ Department of Clinical Epidemiology Leiden University Medical Center Leiden The Netherlands; ^10^ Department of Pediatric Hematology Erasmus University Medical Center‐Sophia Children's Hospital Rotterdam The Netherlands; ^11^ Department of Molecular Hematology, Sanquin Research and Landsteiner Laboratory, Amsterdam University Medical Center University of Amsterdam Amsterdam The Netherlands; ^12^ Department of Experimental Vascular Medicine, Amsterdam University Medical Center University of Amsterdam Amsterdam The Netherlands

**Keywords:** bleeding disorders, platelet activation, platelet factor 4, VWD, VWF

## Abstract

Von Willebrand disease (VWD) is a bleeding disorder caused by quantitative (type 1 or 3) or qualitative (type 2A/2B/2M/2N) defects of circulating von Willebrand factor (VWF). Circulating VWF levels not always fully explain bleeding phenotypes, suggesting a role for alternative factors, like platelets. Here, we investigated platelet factor 4 (PF4) in a large cohort of patients with VWD. PF4 levels were lower in type 2B and current bleeding phenotype was significantly associated with higher PF4 levels, particularly in type 1 VWD. Based on our findings we speculate that platelet degranulation and cargo release may play a role across VWD subtypes.

## INTRODUCTION

Von Willebrand disease (VWD) is the most common inherited bleeding disorder, characterized by a deficiency of von Willebrand factor (VWF).^1^ VWF is a large, multimeric protein that interacts with many haemostatic components, collagen, surface receptors on platelets, coagulation factor VIII (FVIII) and other ligands.^2^ VWD is classified in subtypes based on VWF defects that are quantitative (reduction of VWF levels in type 1; or absence of VWF in type 3) or qualitative (in type 2 VWD). Type 2 VWD can be further subdivided into: defects in multimerization or enhanced proteolytic cleavage by ADAMTS13 (Type 2A), enhanced interaction with platelet GPIb (Type 2B), defective binding to platelet GPIb or collagen (Type 2M) or defective binding and stabilization of FVIII (Type 2N). This classification is based on diagnostic assays that determine function and quantity of VWF in the circulation.^1^

However, VWD patients with similar circulating VWF levels can have variable bleeding phenotypes,^3^, ^4^ suggesting that additional disease modifiers, such as release of VWF and other haemostatic cargo from platelets may be of importance in VWD. Platelet factor 4 (PF4) is a platelet‐specific chemokine that can be sensitively measured in plasma after platelet cargo release. This cargo release from platelet alpha granules, or degranulation, is part of the platelet activation process.^5^

In the current study, our aim is to explore the degree of platelet degranulation across a large population of VWD patients. We hypothesize that platelet degranulation could be an additional determinant of the bleeding phenotype in VWD. We first quantified PF4 levels across VWD subtypes and in relation to VWF parameters. Next, we investigated whether PF4 levels are associated with the bleeding phenotype in VWD patients.

## METHODS

### Patients

Adult patients with VWD were included in the ‘Willebrand in the Netherlands’ (WiN) study.^3^, ^6^, ^7^ Patients were included in 2007–2009 and citrated plasma samples were stored at time of inclusion at −80°C. Inclusion criteria were: (1) haemorrhagic symptoms or family history of VWD; and (2) one historically lowest measured value of VWF antigen (VWF:Ag) or ristocetin cofactor activity ≤0.3 IU/ml and/or FVIII coagulant activity (FVIII:C) ≤0.40 IU/ml (for type 2N VWD). Exclusion criteria were treatment with blood products prior to inclusion (<72 h) and pregnancy. The study was in accordance with the Declaration of Helsinki and approved by the Medical Ethical committees of the participating centres. Written informed consent was obtained from all study participants.

### Clinical data and bleeding phenotype

Clinical parameters in the WiN study have been described previously.^3^, ^6^, ^7^ Bleeding scores were assessed using the self‐administered version of the condensed Tosetto bleeding score, while current bleeding phenotype was defined as bleeding episodes that required haemostatic treatment in the year prior to inclusion.^8^, ^9^

Platelet counts of type 2B patients were collected from patient files. Thrombocytopenia was defined as platelet counts below 150 × 10^9^/L. Persistent thrombocytopenia was defined as thrombocytopenia throughout the total follow‐up period based on available platelet count data in medical files. Intermittent thrombocytopenia was defined as platelet counts that fluctuated between reference values 150–400 × 10^9^/L and below 150 × 10^9^/L.

### Plasma measurements and statistics

Measurements of VWF parameters and PF4 in plasma, as well as statistics are described in the Supporting information.

## RESULTS

### PF4 levels are lower in type 2B VWD

**FIGURE 1:**
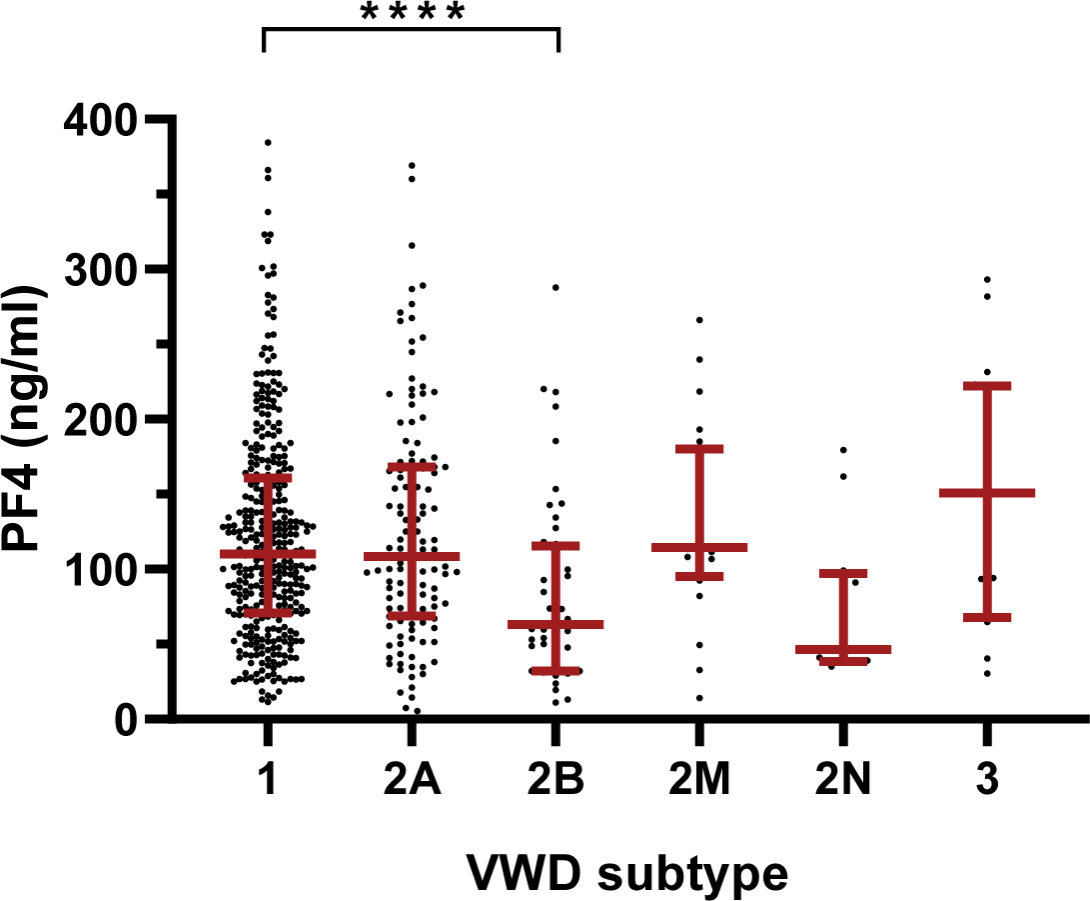
Platelet factor 4 (PF4) levels in the von Willebrand disease (VWD) cohort. Distribution of PF4 plasma levels in ng/ml are shown per subtype of VWD. Subtypes are type 1 (*n* = 368), type 2A (*n* = 125), type 2B (*n* = 50), type 2M (*n* = 20), type 2N (*n* = 12) and type 3 (*n* = 19). Data shown as median ± interquartile range. ****, *p* < 0.0001 [Colour figure can be viewed at wileyonlinelibrary.com]

Plasma PF4 was measured in a total of 594 VWD patients (Table S1; Figure S1). We found that PF4 levels differed across VWD su]btypes (*p* < 0.0001, Figure 1). PF4 levels in type 2B VWD patients [63.2 (32.1–115.4) ng/ml] were lower compared to type 1 VWD patients [110 (70.7–160.7) ng/ml, *p* = 0.0003].

### Potential determinants of PF4 plasma levels in type 2A and 2B VWD

In the total VWD population, we found no association between PF4 and VWF:Ag, VWF activity, or VWF collagen binding (data not shown). We found a small negative correlation between PF4 levels and VWF propeptide/VWF:Ag ratio in VWD patients (*r* = −0.083, *p* = 0.043) which was primarily attributable to type 2A patients (*r* = −0.214, *p* = 0.017). In line with this observation, we found a positive correlation between PF4 and VWF:Ag levels in type 2A VWD patients (*r* = 0.229, *p* = 0.010).

A further, explorative, analysis of PF4 levels in types 2A and 2B showed that levels may be associated with specific mutations (Figure S2A,B). We found significantly lower PF4 levels in type 2B patients with persistent thrombocytopenia when compared to patients with intermittent thrombocytopenia or normal platelet counts (Figure S2C).

### PF4 levels associate with bleeding requiring treatment in VWD patients

**FIGURE 2:**
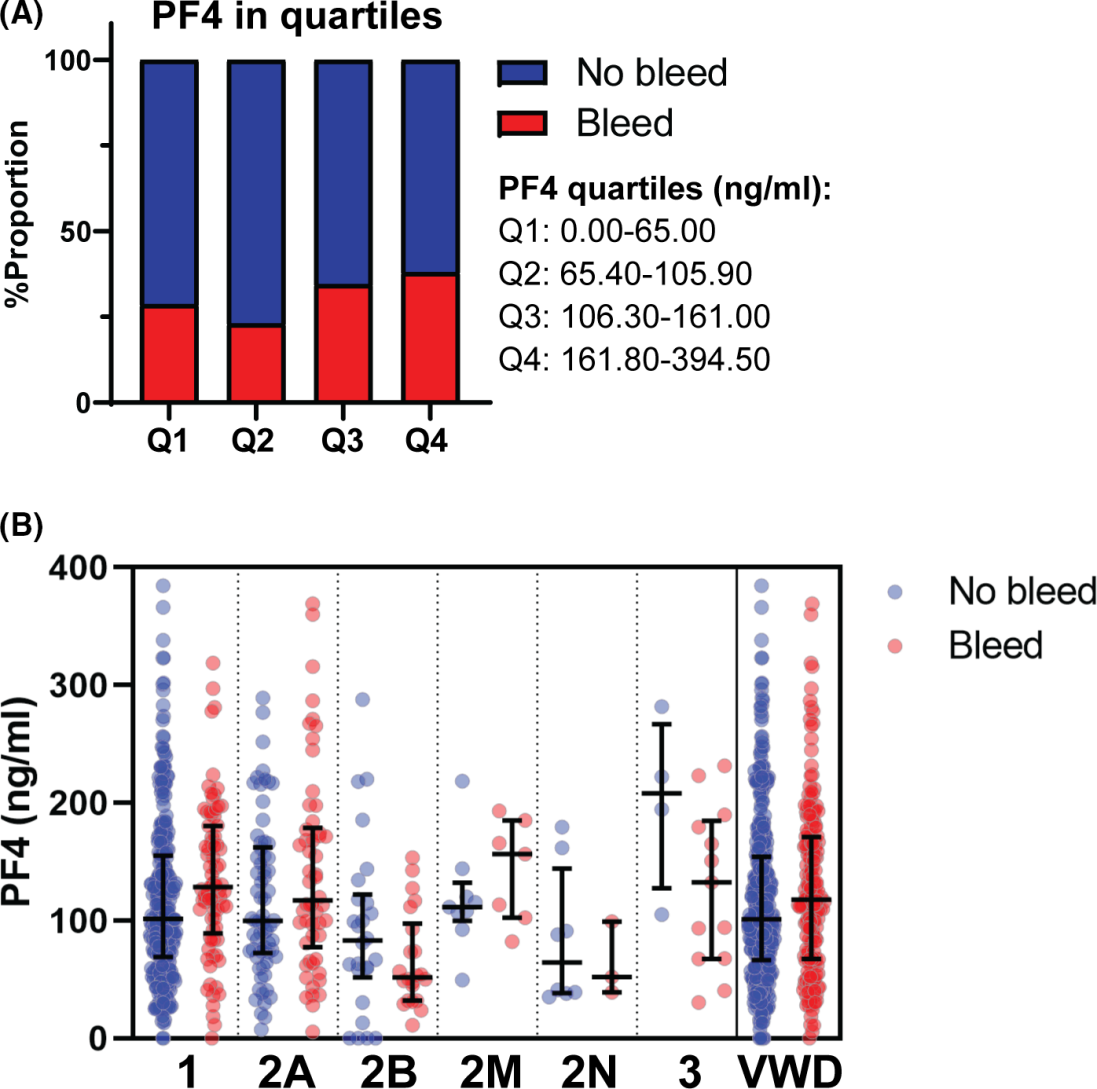
Platelet factor 4 (PF4) levels in relation to current bleeding phenotype in the von Willebrand disease (VWD) cohort. PF4 plasma levels were related to bleeding that required treatment in the year prior to inclusion. For this analysis, PF4 was subdivided into quartiles: 0.00–65.00 ng/ml (Q1), 65.40–105.90 ng/ml (Q2), 106.30–161.00 ng/ml (Q3) and 161.80–384.50 ng/ml (Q4). The proportion of patients with no bleeding (no bleed, blue) versus bleeding (bleed, red) were plotted across the four quartiles of PF4 levels (A). Bleeding versus PF4 levels are also plotted per subtype and total population (B). Data are shown as proportion (A) and median ± interquartile range (B) [Colour figure can be viewed at wileyonlinelibrary.com]

Finally, we investigated whether PF4 levels were associated with bleeding phenotype in VWD patients (Figure 2A). PF4 was not associated with total bleeding score [β = 0.02 (−0.52;0.56), *p* = 0.940]. However, we found that PF4 levels in the total VWD cohort were positively associated with the current bleeding phenotype [overall response (OR) 1.21 (1.02;1.43), *p* = 0.029]. Similarly, the third and fourth PF4 quartiles contained a higher proportion of patients who experienced recent bleeding (Q1 = 28.7%, Q2 = 23.1%, Q3 = 34.5%, Q4 = 38.0%; Figure 2A).

When VWD types were analysed separately we only found a significant association between PF4 and current bleeding phenotype in patients with type 1 VWD [OR 1.40 (1.10–1.79), *p* = 0.007]. Descriptive data are further shown in Figure 2B.

## DISCUSSION

In this study, we measured PF4 levels in a large and well‐defined cohort of VWD patients aiming to investigate platelet degranulation in VWD. We found that PF4 levels were positively associated with the current bleeding phenotype, mainly in type 1 VWD patients. We also identified that type 2B VWD patients, particularly those with persistent thrombocytopenia, had lower PF4 levels in comparison to type 1 VWD patients. Associations between PF4, VWF levels and VWF mutations suggest that platelet degranulation could play role in type 2A and 2B VWD. In conclusion, our associative findings highlight a potential association between PF4 levels, as a measure for platelet degranulation, and current bleeding phenotype in VWD patients.

The role of platelets and their cargo release in the pathophysiology and phenotype of VWD patients is largely unknown, as there have been no large cohort association studies until now. We found that PF4 levels were positively associated with a current bleeding phenotype, particularly in type 1 VWD. Considering that bleeding in type 1 VWD patients is also determined by factors other than VWF,^3^, ^4^, ^10^, ^11^ our data suggest that platelet cargo release may be of particular interest in this subtype. Elevated PF4 levels may be indicative of partial pre‐release of platelet cargo or constitutive platelet activation, suggesting platelets may no longer fully function during primary haemostasis. This might explain the increasing bleeding tendency in patients with higher PF4 levels, but mechanistic studies are required to further elucidate this.

We also found that PF4 levels were lower in type 2B compared to type 1 VWD, especially in those with persistent thrombocytopenia. Possibly, this indicates that the continuous consumption of platelets^12^ (capable of releasing PF4) in some of these patients leads to lower PF4 levels. Platelet degranulation itself could also be affected, as PF4 levels in type 2B patients without thrombocytopenia were also lower than in type 1 VWD. A final explanation for lower PF4 levels in type 2B may be due to megakaryocytic defects in type 2B patients, which could directly affect PF4 synthesis.^13^

In the current study, we also identified a positive correlation between PF4 and VWF levels in type 2A VWD patients. A recent *in vitro* study has demonstrated that VWF and PF4 may interact under specific conditions, possibly at the A2 domain of VWF, and that this interaction may affect ADAMTS13‐mediated cleavage of VWF.^14^ Intriguingly, we found that type 2A patients with A2 mutations had high PF4 levels, which might indicate a similar interaction *in vivo*. We did not find an association between ADAMTS13 activity and PF4 levels (data not shown), but the assay for ADAMTS13 activity is not suited to determine how PF4 affects ADAMTS13‐mediated VWF proteolysis.^15^ Thus, our data cannot decisively answer if PF4 plays a role in VWF interactions and ADAMTS13‐mediated proteolysis *in vivo* yet, but suggests this could ultimately be relevant to type 2A patients.

One limitation of the current study was that platelet degranulation was measured based solely on a plasma marker. Ideally, a parallel approach would also measure platelet cell surface activation markers, but this requires fresh platelet samples. Finally, another limitation is that we had no access to a healthy control dataset that was matched to the patient population. A prospective follow‐up study that includes both VWD and healthy subjects would be very useful to further elucidate the role of platelet degranulation in VWD.

In conclusion, we evaluated PF4 plasma levels as a marker of platelet degranulation in a large cohort of VWD patients. Our findings suggest that platelet degranulation may be associated with current bleeding phenotype in type 1 VWD, and VWF levels and mutations in type 2A and 2B VWD. Further mechanistic and prospective studies on the role of platelet cargo in the pathophysiology of VWD will be needed to elucidate the associations generated in this study.

## CONFLICT OF INTEREST

F. W. G. Leebeek received research support from CSL Behring and Shire for performing the Willebrand in the Netherlands (WiN) study, and is consultant for uniQure, Biomarin, Novo Nordisk and Shire, of which the fees go to the institution. F. Atiq received the CSL Behring‐Heimburger Award 2018, and a travel grant from Sobi. I. van Moort received the CSL Behring‐Heimburger Award 2021. A. J. G. Jansen received speaker fees and travel cost payments from 3SBio, Amgen and Novartis, is on the international advisory board at Novartis and received research support from Sanofi, Argenx and CSL Behring. J. Eikenboom received research support from CSL Behring and he has been a teacher on educational activities of Roche. K. P. M. van Galen received unrestricted research support from CSL Behring and Bayer. J. G. van der Bom has received unrestricted research/educational funding for various projects from the following companies: Bayer Schering Pharma, Baxter, CSL Behring, Novo Nordisk, and Pfizer. In addition, she has been a consultant to Baxter and Pfizer, and she has been a teacher on educational activities of Bayer Schering Pharma. M. H. Cnossen has received unrestricted research/educational and travel funding from the following companies: Pfizer, Baxter, Bayer Schering Pharma, CSL Behring, Novo Nordisk and Novartis, and serves as a member on steering boards of Roche and Bayer of which fees go to the institution. K. Fijnvandraat is a member of the European Haemophilia Treatment and Standardization Board sponsored by Baxter, has received unrestricted research grants from CSL Behring and Bayer, and has given lectures at educational symposiums organized by Pfizer, Bayer and Baxter. K. Meijer received speaker fees from Alexion, Bayer and CSL Behring, fees for participation in trial steering committee for Bayer, consulting fees from Uniqure, and fees for participation in data monitoring and endpoint adjudication committee for Octapharma. S. Schols received travel grants from Bayer and Takeda and consultancy grants from Takeda and Novo Nordisk. None of the other authors has a conflict of interest to declare.

## AUTHOR CONTRIBUTIONS

Maurice Swinkels, Petra E. Bürgisser and Petra E. Bürgisser1 | Iris van Moort performed experiments. Maurice Swinkels and Ferdows Atiq analysed data. Ferdows Atiq retrieved data from patients files. Karina Meijer, Jeroen Eikenboom, Karin Fijnvandraat, Karin P. M. van Galen, Joke de Meris, Saskia E. M. Schols, Johanna G. van der Bom and Marjon H. Cnossen provided essential patient material for the study. Maurice Swinkels, Jan Voorberg, Frank W. G. Leebeek, Ruben Bierings and A. J. Gerard Jansen designed the research and wrote the paper. All authors critically revised and approved of the final version of the manuscript.

## WiN Study group members

Academic Medical Centre, Amsterdam: K. Fijnvandraat, M. Coppens. The Netherlands Haemophilia Society: J. de Meris. Maxima Medical Centre, Eindhoven: L. Nieuwenhuizen. University Medical Centre Groningen, Groningen: K. Meijer, R. Y. J. Tamminga. HagaZiekenhuis, The Hague: P. F. Ypma. Leiden University Medical Centre, Leiden: H. C. J. Eikenboom, J. G. van der Bom, F. J. W. Smiers. Maastricht University Medical Centre, Maastricht: B. Granzen, F. Moenen. Radboud University Medical Centre, Nijmegen: P. Brons, S. E. M Schols. Erasmus University Medical Centre, Rotterdam: F. W. G. Leebeek (principal investigator), M. H. Cnossen, F. Atiq, C. B. van Kwawegen. Van Creveld Clinic, University Medical Centre Utrecht, Utrecht: K. P. M. van Galen.

## Supporting information


**Appendix** S1
