# Supplementary material for: Platelet degranulation and bleeding phenotype in a large cohort of Von Willebrand disease patients
Source: Br J Haematol. 2022 Mar 22;197(4):497–501. doi: 10.1111/bjh.18145 (PMC9314899; doi:10.1111/bjh.18145)
Supplement: Supplementary file 1 — Appendix S1 [file BJH-197-497-s001.docx]

**Supplementary Methods**

Diagnostic laboratory measurements

In the WiN study, plasma levels of VWF antigen (VWF:Ag), VWF activity (measured by VWF monoclonal antibody assay, VWF:Ab), VWF collagen binding capacity (VWF:CB) and FVIII:C were centrally measured in samples obtained at inclusion in the study, at the Erasmus University Medical Centre as described previously (1-3). VWF propeptide (VWFpp) was centrally measured at the Leiden University Medical Centre (Leiden, the Netherlands) (3). VWF multimers and ratios as well as ADAMTS13 activity were assessed as previously described (4, 5).

PF4 plasma levels

To measure PF4 plasma levels, a PF4 ELISA kit (DY795, R&D Systems) was employed according to the manufacturer’s protocol with some adaptations to minimize analytical variation. Between all steps, ELISA plates (Corning, cat#CLS3590) were washed 3x in PBS containing 0.05% Tween-20. Plates were first coated in 2 µg/ml capture antibody in PBS (mouse anti-human PF4) overnight and stored at 4°C until use. Plates were then blocked in PBS + 1% bovine serum albumin (BSA, Sigma, cat#A1470)(PBS/BSA) for 1 hour at RT, after which samples and standard curves were added in duplicate. For standards, we used the recombinant human PF4 provided by the manufacturer (0-4000 pg/ml) as well as a plasma standard based on Normal Pooled Plasma from healthy donors (Sanquin, dilutions ranging from 1:25-1:1600). Each plate had standard curves of both. Patient plasma samples were added in 3 dilutions per sample in PBS/BSA for 2 hours at RT, followed by incubation with 200 ng/ml primary antibody (biotinylated goat anti-human PF4) in PBS/BSA for 2 hours at RT, and finally 1:200 secondary antibody (streptavidin-HRP) in PBS/BSA for 20 min at RT. Ultimately, plates were developed with TMB substrate (Fisher Scientific, cat#10076433) for 5 minutes, stopping the reaction with 2 N H_2_SO_4_. Absorbance was measured on a spectrophotometer (Victor X4, Perkin Elmer) at 480 nm (signal) and 560 nm (background).

PF4 plasma levels in NPP were then determined using the recombinant PF4 standard, by using the aggregate of all plates (n=58) to correct for potential variability across plates. NPP curves were then used to calculate patient PF4 values of the multiple dilutions. Values that did not correspond to a linear section of these plasma curves were omitted.

Statistics

Continuous data are presented as median and interquartile range [IQR], whereas categorical data are presented as number and proportion (%). Normality of data was assessed with four normality tests, which showed that PF4 was not normally distributed. Prior to analysis, outliers were defined as PF4 levels higher than ±2 standard deviations from the mean (Supplemental Figure S1).

Comparison of PF4 levels between VWD types was analyzed using the Kruskal-Wallis test with a post-hoc analysis that was corrected for multiple comparisons using Dunn’s test. Correlation between PF4 and VWF levels was assessed with Spearman’s correlation analysis. Comparisons of mutations and thrombocytopenia in type 2A and type 2B patients were performed using Mann-Whitney U tests as an explorative analysis. The association between PF4 and total bleeding score was analyzed with linear regression analysis, while the association between PF4 and bleeding requiring treatment in the year prior to inclusion in the study was analyzed with binary logistic regression analysis. Regression analyses were adjusted for age, sex and BMI.

Outcomes of linear regression analyses are presented as unstandardized beta (β) and 95% confidence interval (CI), whereas outcomes of logistic regression analysis are presented as odds ratio (OR) and 95%CI. In the linear regression analyses and logistic regression analysis with bleeding as dependent variable, PF4 was proportioned in quartiles. A p-value below 0.05 was considered as significant. All analyses were performed with SPSS version 25 (IBM Statistics).

**Supplementary Figures**

**Supplementary Table 1: Patient characteristics.** Samples from the WiN studies were available from 615 adult VWD patients, where we measured PF4 in a total of 594. Five patients were excluded due to a treatment with VWF concentrate or DDAVP less than 72 hours prior to blood draw, or pregnancy. Sixteen patients were excluded as outliers (PF4 levels >2x SD, Supplemental Figure S1). The largest group was type 1 VWD (n=368), followed by type 2 VWD, which was subdivided in type 2A (n=125), type 2B (n=50), type 2M (n=20) and type 2N (n=12). Finally, a small group had type 3 VWD (n=19)

|  | VWD (n=594) | | | |
| --- | --- | --- | --- | --- |
|  | Total (n=594) | Type 1 (n=368) | Type 2 (n=207) | Type 3 (n=19) |
| Age (years) | 44 [31-58] | 45 [32-57] | 44 [31-59] | 26 [12-54] |
| Female sex | 373 (62.8%) | 249 (67.7%) | 114 (55.1%) | 10 (52.6%) |
| Blood group O | 361 (61.1%) | 252 (69.0%) | 101 (48.8%) | 8 (42.1%) |
| VWF:Ag (IU/ml) | 0.29  [0.17-0.45] | 0.36  [0.21-0.53] | 0.24  [0.16-0.35] | 0.00  [0.00-0.01] |
| VWF:Ab (IU/ml) | 0.22  [0.08-0.53] | 0.43  [0.21-0.70] | 0.08  [0.03-0.15] | 0.00  [0.00-0.00] |
| VWF:CB (IU/ml) | 0.22  [0.07-0.52] | 0.41  [0.19-0.66] | 0.07  [0.06-0.13] | 0.00  [0.00-0.00] |
| FVIII:C (IU/ml) | 0.51  [0.32-0.74] | 0.65  [0.46-0.87] | 0.36  [0.27-0.47] | 0.01  [0.01-0.03] |
| VWFpp/VWF:Ag | 2.79  [1.87-4.96] | 2.17  [1.73-3.35] | 4.51  [3.31-5.91] |  |
| FVIII:C/VWF:Ag | 1.73  [1.38-2.28] | 1.81  [1.47-2.36] | 1.56  [1.24-2.03] |  |
| Bleeding score | 11 [6-17] | 9 [5-15] | 13 [9-20] | 20 [16-27] |
| Bleeding requiring treatment in the year prior to inclusion in the study | 176 (31.1%) | 77 (21.8%) | 86 (44.1%) | 13 (76.5%) |
| Data are presented as median with interquartile range in brackets or number and proportion (%). | | | | |

**Supplemental Figure 1**


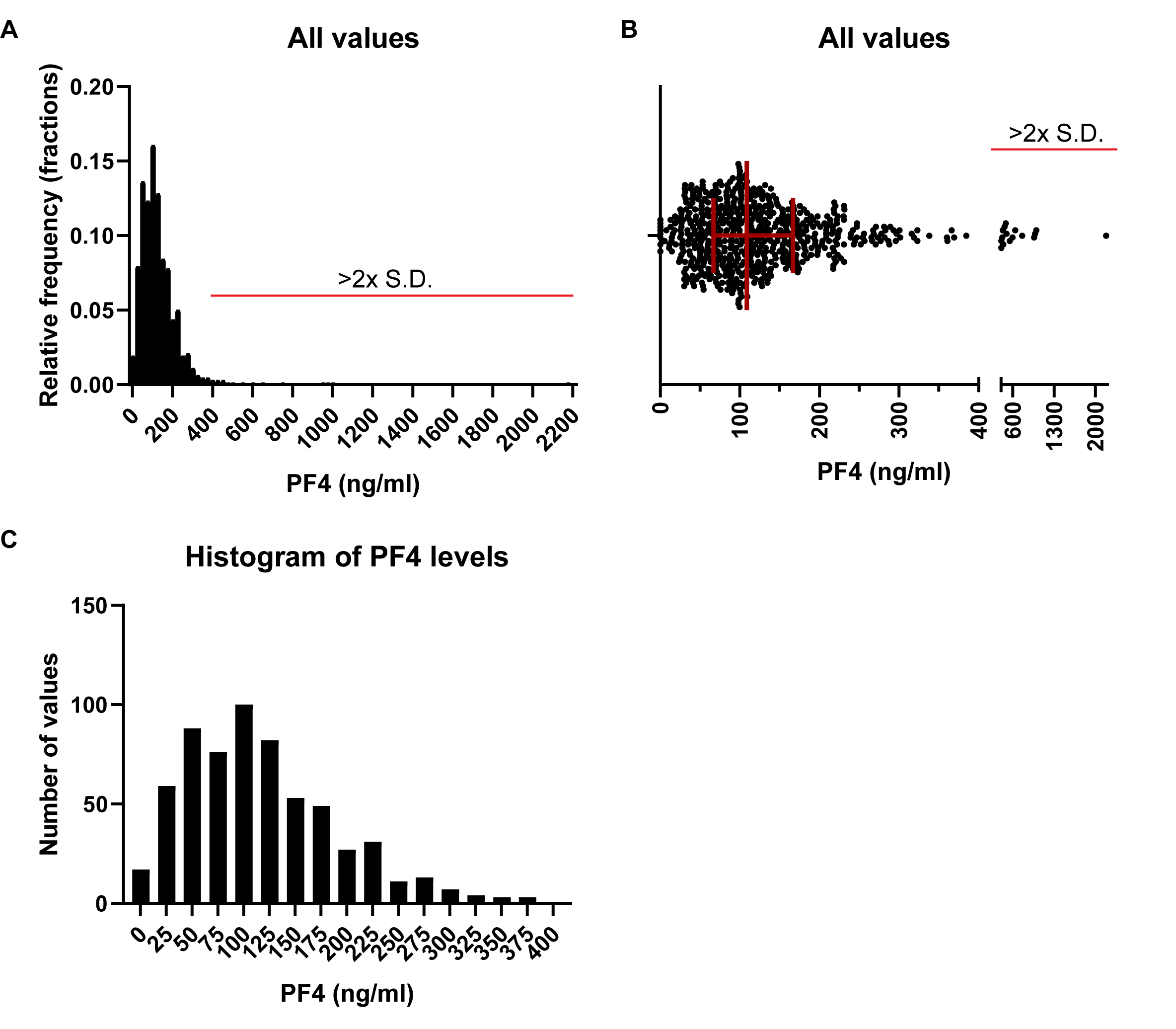


**Supplemental Figure S1:** **Outlier handling and data distribution.** All PF4 data points were analyzed for outliers prior to further analysis, represented as frequency distribution (A) and singular data points (B). Values that were larger than 2x S.D. were excluded (red line), which were 16 data points in total. Data distribution of included PF4 values is also shown (C).

**Supplemental Figure 2**


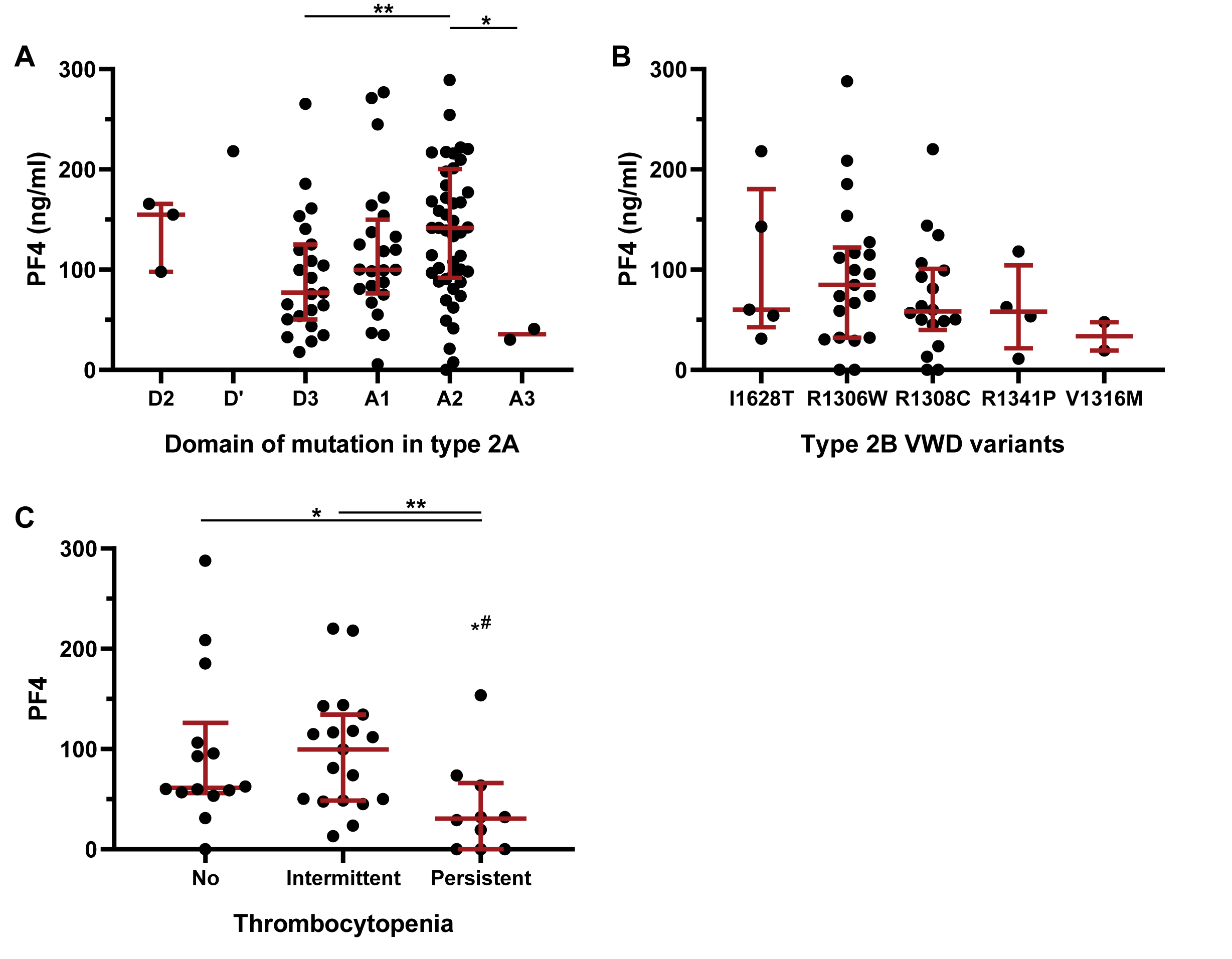


**Supplemental Figure S2: PF4 levels in type 2A and 2B patients.** PF4 levels in VWD patients with different mutations in type 2A- (A) and type 2B patients (B). PF4 levels in type 2B patients with no-, intermittent or persistent thrombocytopenia (C). Data shown as median ± interquartile range. * = p<0.05, ** = p<0.01

**References**

1. de Wee EM, Sanders YV, Mauser-Bunschoten EP, van der Bom JG, Degenaar-Dujardin ME, Eikenboom J, et al. Determinants of bleeding phenotype in adult patients with moderate or severe von Willebrand disease. Thromb Haemost. 2012;108(4):683-92.

2. Sanders YV, Eikenboom J, de Wee EM, van der Bom JG, Cnossen MH, Degenaar-Dujardin ME, et al. Reduced prevalence of arterial thrombosis in von Willebrand disease. J Thromb Haemost. 2013;11(5):845-54.

3. Sanders YV, Groeneveld D, Meijer K, Fijnvandraat K, Cnossen MH, van der Bom JG, et al. von Willebrand factor propeptide and the phenotypic classification of von Willebrand disease. Blood. 2015;125(19):3006-13.

4. Boender J, Nederlof A, Meijer K, Mauser-Bunschoten EP, Cnossen MH, Fijnvandraat K, et al. ADAMTS-13 and bleeding phenotype in von Willebrand disease. Res Pract Thromb Haemost. 2020;4(8):1331-9.

5. Boender J, Atiq F, Cnossen MH, van der Bom JG, Fijnvandraat K, de Meris J, et al. Von Willebrand Factor Multimer Densitometric Analysis: Validation of the Clinical Accuracy and Clinical Implications in Von Willebrand Disease. Hemasphere. 2021;5(3):e542.
